# Benthic macroinvertebrates as indicators of water quality: A case study of estuarine ecosystems along the coast of Ghana

**DOI:** 10.1016/j.heliyon.2024.e28018

**Published:** 2024-03-13

**Authors:** Dorothy Khasisi Lukhabi, Paul Kojo Mensah, Noble Kwame Asare, Margaret Fafa Awushie Akwetey, Charles Abimbola Faseyi

**Affiliations:** a Centre for Coastal Management-Africa Centre of Excellence in Coastal Resilience, University of Cape Coast, Cape Coast 00223, Ghana; b Department of Fisheries and Aquatic Sciences, School of Biological Sciences, University of Cape Coast, Ghana; c Institute for Water Research, Rhodes University, Makhanda 6140, South Africa

**Keywords:** Estuaries, Benthic macroinvertebrates, Ecosystem health, Pollution, Water quality

## Abstract

Increasing human activities in coastal areas of Ghana have led to the degradation of many surface waterbodies, with significant consequences for the ecosystems in the affected areas. Thus, this degradation extremely affects the health of ecosystems and disrupts the essential services they provide. The present study explored the use of benthic macroinvertebrates as an indicator of estuarine degradation along the coast of Ghana. Water and sediment samples were collected bimonthly from Ankobra, Kakum and Volta estuaries for physicochemical parameters, nutrients and benthic macroinvertebrates. The findings revealed the dominance of pollution-tolerant taxa such as *Capitella* sp., *Nereis* sp., *Heteromastus* sp., *Tubifex* sp., *Cossura* sp. and *Chironomous* sp. in Kakum Estuary while pollution-sensitive taxa such as *Scoloplos* sp., *Euridice* sp., *Lumbriconereis* sp. and *Pachymelania* sp. in the Volta Estuary. The species-environment interactions showed dissolved oxygen, temperature, salinity, orthophosphate, nitrates, ammonium, electrical conductivity, turbidity, and chemical oxygen demand as the most significant parameters that complement the use of benthic macroinvertebrates as indicators of environmental quality in the studied estuaries. There were correlations of some benthic macroinvertebrate taxa with environmental factors in the estuaries suggesting low, moderate and high levels of pollution in the Volta, Kakum and Ankobra estuaries, respectively. Nevertheless, the study finds Kakum Estuary to be the ecologically healthiest estuary than the Volta and Ankobra Estuaries. Therefore, the study has shown benthic macroinvertebrates as a key indicator of ecosystem health alterations, and it is recommended that they should be incorporated with other environmental data for pollution monitoring in Ghanaian coastal waters.

## 1 Introduction

Estuaries are semi-enclosed areas of water that are openly connected to the ocean and have a combination of freshwater and saltwater features [1,2]. These ecosystems, being among the world's most productive, play a crucial role in supporting diverse life forms, including fish, shellfish, migratory birds, benthic organisms, and aquatic plants [[3], [4], [5], [6]]. The distribution, and abundance of these life forms are influenced by biophysical forces such as tides, waves, and temperature [7]. Estuaries provide essential ecosystem services with provisioning, supporting, regulatory, and cultural functions for the environment and humans, resulting in a high dependence on them [8,9].

The health of estuarine ecosystems is evaluated through various indicators and parameters reflecting their overall condition [[10], [11], [12], [13]]. Biological monitoring, specifically biomonitoring, employs bioindicators, such as benthic macroinvertebrates, to assess ecosystem conditions based on organisms' reactions to environmental changes [[14], [15], [16], [17]]. High numerical abundance, high sensitivity to environmental stressors, wide distribution, low mobility, and a high capacity for measurement and standardisation are all desirable qualities for benthic macroinvertebrates as bioindicators [[18], [19], [20]]. Benthic macroinvertebrates, sensitive to environmental changes, serve as valuable tools for monitoring aquatic ecosystem health, being either pollution-sensitive or pollution-tolerant [21,22].

Pollution-sensitive species, like stoneflies, mayflies, caddisflies, flatworms, and leeches, are highly responsive to environmental pollution and are used as bioindicators to assess ecosystem health [[21], [22], [23], [24], [25], [26]]. Their absence or scarcity indicates pollution or environmental degradation [[24], [25], [26]].

On the other hand, pollution-tolerant species, including midge larvae, oligochaeta, scuds, copepods, and snails, thrive in polluted or disturbed aquatic ecosystems [[27], [28], [29], [30]]. While previous studies in Ghana have explored marine benthic macroinvertebrates, more data is needed to monitor coastal waters and regulate anthropogenic activities degrading surface waterbodies [[29], [30], [31], [32], [33], [34], [35]]. Table 1 lists other coastal waterbodies in the country that have previously been assessed.

**Table 1:** Some coastal waterbodies previously assessed for environmental quality in Ghana

Waterbodies	Matrices Assessed	References
Pra Estuary	water, sediment, benthic macroinvertebrates	[34,35,[37], [38], [39]]
Nyan Estuary	water, sediment, benthic macroinvertebrates, fish	[33,40]
Muni Lagoon	water, fish	[41,42]
Whin Estuary	water, sediment, fish	[40,[43], [44], [45]]
Chemu Lagoon	water	[46]
Benya Lagoon	water, sediment, benthic macroinvertebrates, fish	[32,35,47]
Narkwa Lagoon	water, bivalves	[42,43,45,48,49]
Amisa Lagoon	sediment	[50]
Fosu Lagoon	water, sediment, benthic macroinvertebrate, fish	[32,51,52]

The degradation of surface waterbodies is caused by human activities such as mining, illegal fishing, improper waste disposal, open defecation, and the use of harmful chemicals in farming [36].

This study therefore used information on the abundance, composition, and diversity of benthic macroinvertebrates to assess the condition of three notable Ghanaian estuarine ecosystems (i.e., Ankobra, Kakum and Volta), examining their relationship with environmental factors prevailing in the estuaries. Additionally, the study also hypothesised that anthropogenic impacts on environmental factors significantly influenced benthic macroinvertebrates' composition and diversity.

## 2 Materials and Methods

### 2.1 Study area

**Fig. 1:**
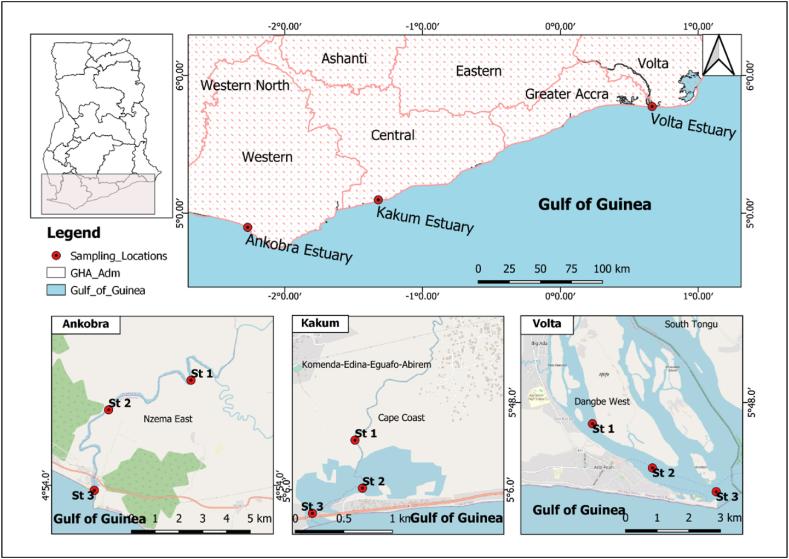
Map of the coastal belt of Ghana showing the study locations. **Note:** St 1-upper reaches, St 2-middle reaches, St 3-lower reaches. Every station represents three sub-stations on a horizontal transect; right, mid and left

The study was carried out in three important estuaries along the coast of Ghana, which is mainly a high-energy coast about 540 km stretching from Aflao (Togo border) to the La Cote D'Ivoire border. Ghana's coastline is divided into three based on geomorphologic characteristics: West, Central and East coasts (Fig. 1) [53]. For this study, three estuaries; Ankobra, Kakum and Volta, from the West, Central and East coasts, respectively, were selected to represent the various sections of the coastline.

#### 2.1.1 Ankobra Estuary

Ankobra Estuary lies between latitudes 4°55′N and 4°54′N, and longitudes 2°17′W and 2°15′W. It is approximately 10 km within the mangrove ecosystems and discharges into the Gulf of Guinea at Asanta. It is bound to the east by Nzema East district and to the south by the Gulf of Guinea [54]. The Estuary forms part of the Ankobra basin, which has a total surface area of approximately 8400 km^2^ and runs through Dompem, Prestea, Bogoso, Asankragua, Awaso, Tarkwa, Egyembra, Esiama and Axim townships. The main economic activities along the drainage system include both legal and illegal gold mining, cash crop farming and fishing. Illegal mining activities from upstream form the major challenge in this estuary, resulting in massive siltation that threatens the estuarine ecosystem's health and consequently affects its biodiversity. Additionally, surface run-offs from upland agricultural fields, as well as residential and municipal effluents contribute to the pollution of this estuary [55].

#### 2.1.2 Kakum Estuary

It lies between latitudes 5°30′N and 5°47′N, and longitudes 0°12′W and 0°35′W. It is located along the Cape Coast-Takoradi highway near Iture community in the Cape Coast metropolis, Central Region and Central section of the coastline. It is formed by the Kakum River and Sweet River, which drain from a rapidly urbanised area of the region. The major economic activities practised by communities along the estuary include sand winning, salt mining and fishing [56].

#### 2.1.3 Volta Estuary

Volta Estuary lies between latitudes 5°46′N and 5°48′N and longitudes 0°37′E and 0°41′E. The estuary which is about 1.2 km wide is located in the lower basin of River Volta at Ada in Greater Accra Region within the coastal savannah zone and at the discharging point into the sea. It is the largest river basin in Ghana, with a drainage area covering approximately 379,000 km^2^. Volta Estuary quality and quantity are dependent on the Akosombo and Kpong dams built on the river. The Black, White, Red Volta and Oti Rivers constitute the sources of the Volta River, originating from Burkina Faso. Volta River basin cuts across six riparian countries as its catchment and comprises the major sediment supply to the Gulf of Guinea. These include; Burkina Faso (43 %), Ghana (42 %), Togo (15 %), Benin, Cote d’Ivoire and Mali (15 %) [57].

### 2.2 Sample collection

Samples were collected bi-monthly between April 2022 and February 2023 to cover one hydrological year at low tide following a tide table (https://tides4fishing.com/af/ghana). Each estuary was demarcated into three zones; the upper, middle and lower reaches to ensure the collection of representative samples. Zonation of the estuaries was based on proximity to the riverine system using observation of mangroves and distance with the aid of a handheld Global Positioning System (GPS) gadget (Garmi etrex 60).

#### 2.2.1 Water

*In situ* measurements of surface water temperature (SWT), dissolved oxygen (DO), electrical conductivity (EC), salinity and pH were performed using a HORIBA water quality monitor, model U-5000 (JAPAN) with multi-parametric probes. Turbidity and total suspended solids (TSS) were measured using a pre-calibrated multi-parametric photometer (DR 900). Water samples were collected in pre-cleaned 350-ml plastic polyethylene bottles rinsed with deionised water. Before transportation, water samples were kept under ice for laboratory analysis of chemical oxygen demand (COD), nitrate-nitrogen (NO_3_–N), ammonium-nitrogen (NH_4_–N)and orthophosphate (${\mathrm{PO}}_{4}^{3-})$.

#### 2.2.2 Benthic macroinvertebrates

Three replicate samples of benthic macroinvertebrates were collected at each sampling station using Ekman grab (15 cm × 15 cm) at low tide alongside the water samples. Samples were screened in the field using a set of sieves with mesh sizes of 4.0 mm, 2.0 mm and 0.5 mm. During the sieving process, the larger sieves were stacked above the smaller ones and fauna retained on the sieves were preserved in 10 % formalin for further laboratory examination. The preserved samples were stained with eosin dye before sorting to enhance visibility. The benthic macroinvertebrates found were observed under a dissecting microscope and identified to the lowest possible taxonomic level with the aid of relevant manuals and keys [[58], [59], [60], [61]]. The counts of different taxa groups and individual species were recorded for further analysis.

### 2.3 Laboratory analyses

#### 2.3.1 Analysis of physicochemical parameters in water

a. Nitrate-Nitrogen (NO_3_–N) determination

NO_3_–N was determined using UV spectrophotometric method [62]. In this method, 1 ml of hydrochloric acid (HCl) was added to 50 ml of filtered sample and mixed. The absorbance was read against re-distilled water set at zero absorbance. A wavelength of 220 nm was used to obtain NO_3_–N reading, while interference due to dissolved organic matter was obtained by reading the wavelength at 275 nm. To obtain absorbance due to NO_3_–N, the absorbance reading at 275 nm was subtracted from the reading at 220 nm and concentration was calculated using equation (1) (R^2^ = 0.99) obtained from a nitrate-nitrogen standard calibration curve.

1 y=0.1719x

b. Orthophosphate $\left({\text{PO}}_{4}^{3-}\right)$ determination

Orthophosphate was determined using Ascorbic acid method [62]. The prepared reagents of sulphuric acid (A), potassium antimonyl tartrate solution (B), ammonium molybdate solution (C) and ascorbic acid solution (D) were mixed in the ratio of 10:1:3:6, respectively. In 50 ml of the sample, a drop of phenolphthalein indicator was added, together with 8 ml of the combined reagent and mixed. After 10 min of reaction time, the absorbance of each sample was read at 880 nm. A blank reagent was used as the reference. For turbid samples, a sample blank was prepared by adding all reagents except ascorbic acid and potassium antimonyl tartrate to the sample, and the blank absorbance was subtracted from the sample absorbance reading. The concentration of orthophosphate was calculated using equation (2) (R^2^ = 0.99) obtained from the orthophosphate standard calibration curve.

2 y=0.5543x

c. Ammonium-Nitrogen (NH_4_–N) determination

Ammonium-Nitrogen was determined using Nesslerisation method [62]. In this method, 1 ml of ZnSO_4_ solution and 0.4 ml NaOH were added to 100 ml of the sample to obtain a pH of 10.5. This was allowed to settle and a suitable aliquot of the sample (25 ml) was taken after filtration. One (1) drop of EDTA reagent was added, together with 3 ml of Nessler reagent and the contents were thoroughly mixed. A blank was prepared in the same way by using distilled water instead of the sample. Absorbance was read after 10 min at 410 nm from a UV scanning spectrophotometer, then used to calculate NH_4_–N concentration using equation (3) (R^2^ = 0.98) obtained from the ammonium-nitrogen standard calibration curve.

3 y=0.0023x

d. Chemical oxygen demand (COD) determination

Chemical oxygen demand was determined on unfiltered samples by dichromate oxidation using the closed reflux, titrimetric method according to Standard Methods for Examination of Water and Wastewater [62]. For this analysis, standard 10 ml borosilicate ampules were used as digestion vessels while standard potassium dichromate was used as the digestion solution. The ampules were washed with 20 % H_2_SO_4_ before use to prevent contamination. Volumetric measurements were made in the order of 2.5 ml of the sample, 1.5 ml of digestion solution and 3.5 ml of sulphuric acid reagent. The sample was placed in the ampule and the digestion solution was added. The sulphuric acid reagent was carefully run down the inside of the ampules so an acid layer was formed under the sample-digestion solution layer. The ampules were tightly capped and inverted several times each for homogenisation. The ampules were placed in a block digester pre-heated to 150 °C and refluxed for 2 h behind a protective shield then allowed to cool to room temperature. Using standardised 0.01 M ferrous ammonium sulphate (FAS) solution and 2–3 drops of ferroin indicator, the mixture was titrated until the end point was achieved. In the same manner, a blank containing the reagents and a volume of distilled water equal to that of the sample was refluxed and titrated. Chemical Oxygen Demand was calculated using the formula in equation (4).

4 $$\text{COD}\;\left(\text{mg}/l\right)=\frac{\left(A-B\right)X\;M\;X\;8000}{ml\;sample}$$

where:

A = volume (ml) of FAS used for blank, B = volume (ml) of FAS used for sample, M = molarity of FAS used, 8000 = milliequivalent weight of oxygen x 1000 ml/l.

#### 2.3.2 Analysis of nutrients in sediments

a. Nitrate-Nitrogen and orthophosphate

Nitrate-Nitrogen and orthophosphate in the estuarine sediments were first extracted using calcium sulphate and Mehlich-2 extraction methods for nitrogen and orthophosphate respectively, using the Hach method [63]. After extraction, the filtrate was subjected to UV spectrophotometric and Ascorbic acid methods [62], respectively.

#### 2.3.3 Statistical analyses of data

Descriptive statistics were carried out to summarise physicochemical data and results were presented in a table and graphs. The differences in median concentrations of physicochemical parameters in the estuaries were tested using One-way non-parametric ANOVA (Kruskal-Wallis test). To check whether the residuals followed a normal distribution, the Shapiro-Wilk test was applied. In case of significant differences among groups, Tukey's post hoc test was performed. Descriptive statistics, ANOVA and normality tests were carried out in SigmaPlot software (v.14.0) with a significance threshold set at α = 0.05. Macroinvertebrate structure was described through the species richness (d), the Shannon-Wiener diversity index (H′), and evenness (J′). Richness was determined from Margalef's index (d) calculated as d = $\frac{s-1}{\ln\;N}$ where *s* is the number of species in a sample and *N* is the number of individuals in the sample. Species diversity was calculated using the equation given as H’ = $-\sum_{i=1}^{s}Pi\;\left(\ln\;Pi\right)$ where *Pi* is the proportion of the ith species and *s* is the number of species in a sample. Evenness was determined using Pielou's index calculated as J’ = $\frac{H^{\prime }}{\left(\ln\;s\right)}$ where *s* is the number of species and H′ the Shannon-Wiener diversity index. Spearman's rank order correlation analysis was used to establish the relationship between physicochemical parameters and macroinvertebrate abundance which was performed in Palaeontological Statistics (PAST) software (v. 4.03).

## 3 Results

### 3.1 Spatial variation in physicochemical parameters in the study sites

The statistical summary of physicochemical characteristics of the estuaries is shown in Table 2. There was variation in median SWT in the estuaries over the study period (P=<0.001; H = 28.9). The SWT in Volta Estuary (29.7 °C) differed significantly from that recorded in both Kakum (27.9 °C) and Ankobra Estuaries (27.6 °C), (P < 0.01). However, there was no significant difference in SWT in Ankobra and Kakum Estuaries (P = 1.0).

**Table 2:** Statistical summary of physicochemical characteristics of water and sediments in the selected estuaries

	Estuaries		
Parameter	Ankobra	Kakum	Volta
Temp (°C)	(27.6 ± 1.4) ^**b**^	(27.9 ± 1.4) ^**b**^	(29.7 ± 1.3) ^**ab**^
	**25.9**–**30.1**	**24.4**–**29.6**	**27.1**–**31.9**
DO (mg/l)	(5.4 ± 2.7)^a^	(5.6 ± 2.4) ^**a**^	(6.1 ± 0.9) ^**a**^
	**0.0**–**10.9**	**0.23–9.4**	**4.4**–**9.2**
pH	(6.2 ± 2.4) ^**a**^	(5.0 ± 2.3)^a^	(6.4 ± 2.2) ^**a**^
	**0.9–8.7**	**0.7**–**8.9**	**1.2**–**8.8**
EC (μs/cm)	(93.0 ± 7004.4) ^**a**^	(7580.0 ± 12516.11) ^**b**^	(1420.0 ± 4648.4) ^**c**^
	**48.0**–**29800.0**	**160.0**–**40500.0**	**80.0**–**29900.0**
Salinity (ppt)	(0.1 ± 4.7) ^**a**^	(0.3 ± 6.7) ^**b**^	(0.4 ± 2.9) ^**b**^
	**0.0**–**20.6**	**0.0**–**20.7**	**0**–**19.5**
Turbidity (NTU	(751.5 ± 940.4) ^**a**^	(44.5 ± 32.2) ^**b**^	(5.0 ± 7.1) ^**c**^
	**47.3**–**3292.0**	**11.0**–**151.3**	**0.0**–**41.0**
w. Nitr (mg/l)	(1.5 ± 3.9) ^**a**^	(2.3 ± 1.1) ^**a**^	(0.9 ± 0.4) ^**b**^
	**0.0**–**21.8**	**0.0**–**4.9**	**0.0**–**2.1**
w. Orth (mg/l)	(11.0 ± 8.9)^a^	(2.7 ± 5.6) ^**b**^	(1.5 ± 5.3) ^**a**^
	**0.2**–**54.3**	**0.4**–**26.1**	**0.0**–**22.8**
Amm (mg/l)	(52.9 ± 135.0) ^**a**^	(38.8 ± 45.1) ^**a**^	(53.3 ± 57.2) ^**a**^
	**15.2**–**658.3**	**3.9**–**161.9**	**0.87**–**277.8**
COD (mg/l)	(2160 ± 8725.6) ^**a**^	(474.0 ± 1592.3) ^**b**^	(230.4 ± 1954.8) ^**b**^
	**0.0**–**56160.0**	**0.0**–**8480.0**	**0.0**–**6960.0**
S. Nitr (mg/l)	(1.8 ± 59.6) ^**a**^	(4.1 ± 14.7) ^**b**^	(6.6 ± 3.4) ^**a**^
	**0.3**–**299.9**	**0.0**–**67.8**	**0.0**–**10.7**
S. Orth (mg/l)	(3.2 ± 21.2) ^**a**^	(18.1 ± 21.4) ^**b**^	(6.6 ± 24.1) ^**a**^
	**0.0**–**81.2**	**2.2**–**81.8**	**0.0**–**100.0**

**Note:** The numbers in parenthesis are values for median and standard deviations, while those in bold represent the range. On each row, the medians with the same superscript letter are **not** significantly different at p = 0.05 level; n = 54 while those with different superscript letters indicate significant differences. **Abbreviations:** Temp-Temperature, DO-dissolved oxygen, EC-electrical conductivity, w. Nitr-nitrate-nitrogen concentration in water, w. Orth-orthophosphate concentration in water, Amm-ammonium-nitrogen, COD-chemical oxygen demand, S. Nitr-nitrate-nitrogen concentration in sediments, and S. Orth-orthophosphate concentration in sediments.

Moreover, the three estuaries did not vary significantly in DO concentration and pH, with the Kruskal-Wallis test revealing P > 0.04 and 0.08 for DO and pH respectively.

The highest and lowest median concentrations of EC were recorded in Kakum (7580 μS/cm) and Ankobra Estuaries (93.0 μS/cm), respectively. The EC concentration in the estuaries varied significantly (P < 0.001; H = 50.4). The median concentration of EC in Kakum Estuary was statistically different from both Ankobra and Volta Estuaries (P < 0.001) whereas the median EC concentration in the Volta Estuary (1420 μS/cm) differed significantly from Ankobra Estuary (P = 0.004).

The highest and lowest median turbidity values were recorded in Ankobra (751.5 NTU) and Volta Estuaries (5.0 NTU), respectively. Turbidity in the estuaries varied considerably, ranging from 0.0 NTU in the Volta Estuary to above 3000 NTU in Ankobra Estuary, however, turbidity differed significantly across the three estuaries (P < 0.001).

Generally, nitrate-nitrogen concentrations in the sediments was relatively higher than in the water across the three estuaries, with maximum concentration (300 mg/L) recorded in Ankobra Estuary. Nitrate-nitrogen concentration in sediments in Kakum Estuary differed significantly (P < 0.001; H = 48.0) from that of both Volta (P < 0.001) and Ankobra Estuaries (P < 0.06). However, no significant differences were observed between the two waterbodies (P = 0.747). Additionally, in the water column, Ankobra Estuary also recorded the highest concentration of 21.8 mg/L and there was no significant difference between Ankobra and Kakum Estuaries (P = 0.326). Significant differences in nitrate-nitrogen concentration were observed between the Volta Estuary and both the Kakum and Ankobra Estuaries (P < 0.001). Among all the nutrients assessed, ammonium-nitrogen had the highest concentration in the water, reaching over 650 mg/L in Ankobra Estuary with no significant difference from the other two estuaries (P = 0.279; H = 2.557).

A relatively high range of orthophosphate concentration (0–100 mg/L) was recorded in the sediments in comparison to the water. Orthophosphate concentration differed significantly between Kakum and Ankobra Estuaries as well as between Kakum and Volta Estuaries (P ≤ 0.001) in both the water column and sediment matrices.

The highest and lowest COD concentrations were recorded in Ankobra (2160 mg/L) and Volta (230 mg/L). There were variations in COD concentrations across the estuaries (P=<0.001; H = 32.0). The concentration of COD in Ankobra differed significantly from that recorded in both Volta and Kakum Estuaries (P=<0.001). However, no significant difference occurred in COD concentrations between Kakum and Volta Estuaries (P = 0.4).

### 3.2 Occurrence of benthic macroinvertebrates

During the sampling period, 26 taxa belonging to 24 families and six classes were recorded in the estuaries (see the summary in Table 3). In the Kakum Estuary, six classes were identified including polychaeta, crustacea, oligochaeta, clitellata, and insecta. Polychaetes were the most dominant group, while *Chironomous* sp. was the most abundant species in the estuary (see Table 3 for further details).

**Table 3:** Occurrence of benthic macroinvertebrates in the selected estuaries

Class	Family	Taxa	Number of Individuals		
			Kakum	Volta	Ankobra
**Polychaeta**	Capitellidae	*Capitella* sp.	33	157	2
	Capitellidae	*Notomastus* sp.	43	1	–
	Capitellidae	*Heteromastus* sp.	42	–	–
	Nereididae	*Nereis* sp.	55	34	3
	Orbiniidae	*Scoloplos* sp.	24	63	–
	Pilargidae	*Sigambra* sp	113	3	–
	Nephtydae	*Nephtys* sp.	131	9	2
	Scalibregmatidae	*Polyphysia* sp.	12	–	1
	Lumbrineridae	*Lumbriconereis* sp.	–	8	–
	Syllidae	*Syllis* sp.	–	3	–
	Cossuridae	*Cossura* sp.	1	–	–
	Phyllodocidae	*Phyllodoce* sp.	2	–	–
	Lopadorrhynchidae	*Lopadorrhynchus* sp.	9	–	–
**Clitellata**	Glossiphoniidae	*Glossiphonia* sp.	1	–	–
**Oligochaeta**	Naididae	*Tubifex* sp.	9	3	1
**Crustacea**	Mysidae	*Mysis* sp.	–	1	–
	Penaeidae	*Penaeus* sp.	–	4	154
	Aoridae	*Bemlos* sp.	146	4	–
	Gammaridae	*Gammarus* sp.	26	–	–
	Ocypodidae	*Uca* sp.	1	–	1
	Cirolanidae	*Eurydice* sp.	–	34	–
	Maeridae	*Elasmopus* sp.	5	–	–
	Coenobitidae	*Coenobita* sp.	–	1	–
**Insecta**	Chironomodae	*Chironomous* sp.	169	1	–
**Gastropoda**	Hemisinidae	*Pachymelania* sp.	–	150	–
	Potaminidae	*Tympanotonus* sp.	–	43	–

**Note**: Numbers are total number of individuals; - indicates absent.

Five classes were found in the Volta Estuary: polychaeta, crustacea, gastropoda, oligochaeta, and insecta. Polychaetes were the dominant species, with *Capitella* sp. being the most abundant. Moreover, the crustaceans in the Volta Estuary were *Eurydice* sp., *Penaeus* sp., *Bemlos* sp., *Mysis* sp. and *Coenobita* sp. (Table 3). Overall, the Ankobra Estuary had the fewest benthic macroinvertebrates. The most abundant organism was *Penaeus* sp. and other macroinvertebrates encountered in low abundances included *Uca* sp., *Tubifex* sp., *Polyphysia* sp., *Nephtys* sp. and *Capitella* sp.

### 3.3 Composition of benthic macroinvertebrates

**Fig. 2:**
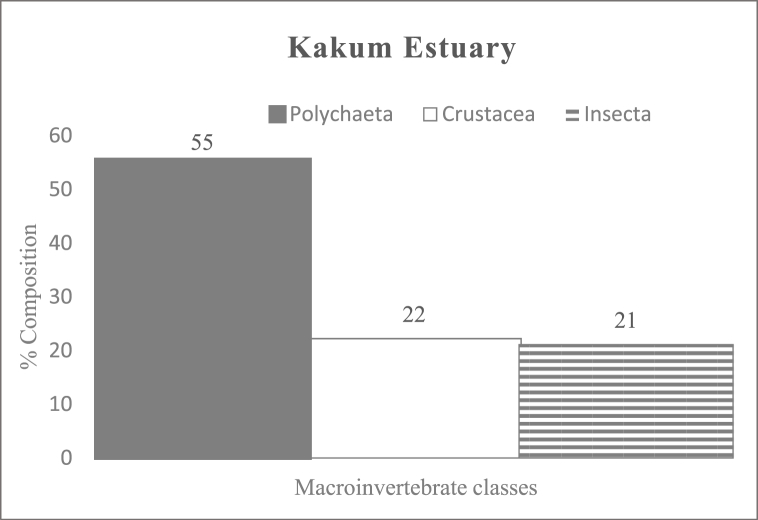
Percentage composition of macroinvertebrate classes in Kakum Estuary

**Fig. 3:**
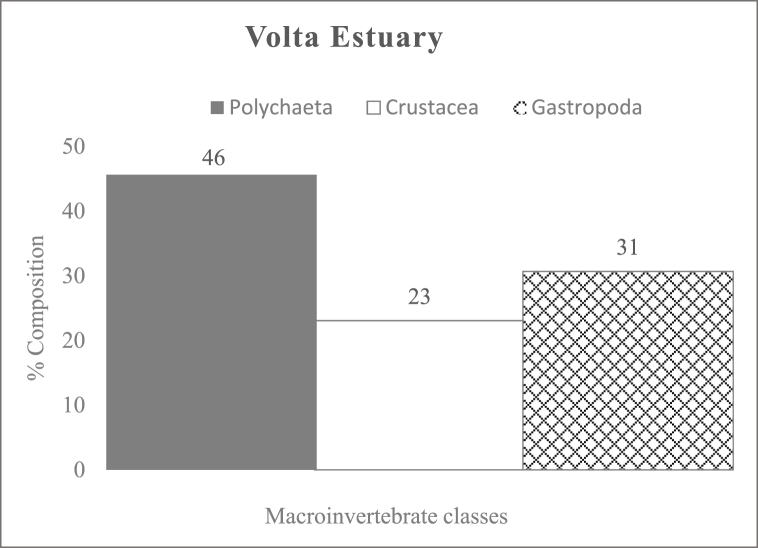
Percentage composition of macroinvertebrate classes in Volta Estuary

**Fig. 4:**
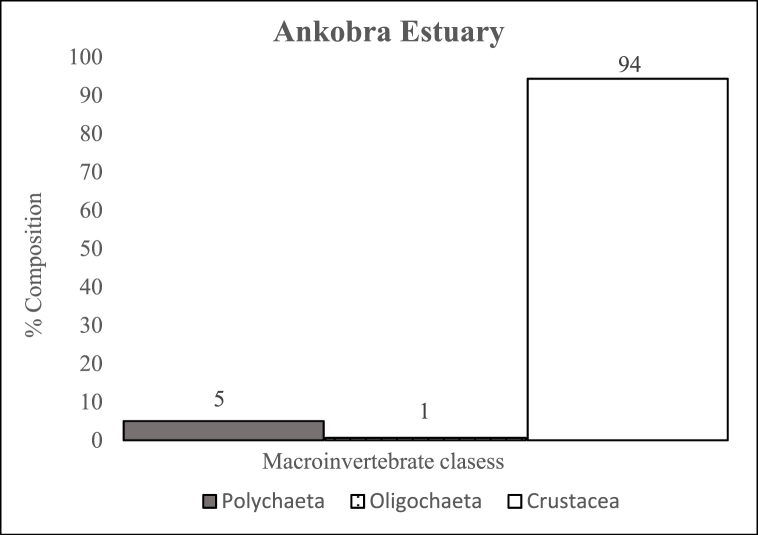
Percentage composition of macroinvertebrate classes in Ankobra Estuary

Polychaetes were the most dominant macroinvertebrates in the estuaries. In Kakum (Fig. 2) and Volta (Fig. 3) Estuaries, they accounted for 55 % and 46 % of the total macroinvertebrate fauna recorded. Whereas, in Ankobra Estuary (Fig. 4), crustaceans were the most dominant group represented by *Penaeus* sp., which accounted for 94 % of all the benthic fauna encountered. In this study, a number of benthic faunas were estuary specific, for instance, *Phyllodoce* sp. (Polychaetes), *Elasmopus* sp. (Crustaceans) in the Kakum Estuary and *Pachymelania* and *Tympanotonus* sp. (Gastropods), *Mysis* sp. (Crustacean) in the Volta Estuary.

### 3.4 Spatial variation in diversity of macroinvertebrates in the selected estuaries

**Fig. 5:**
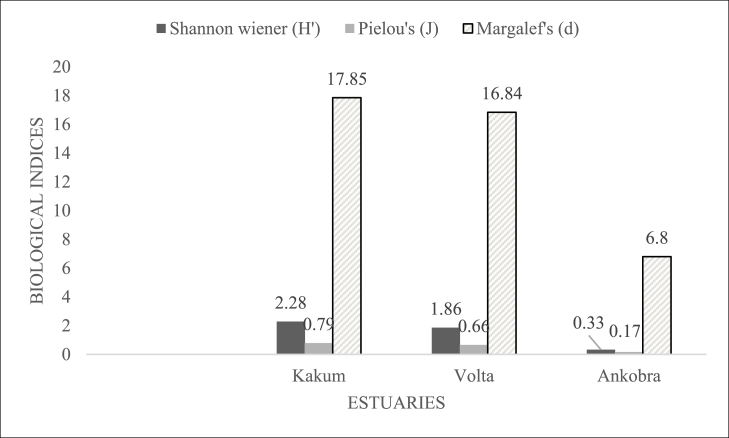
Diversity indices of benthic macroinvertebrates in the (a) Kakum and (b) Volta and (c) Ankobra Estuaries

The number of specimens, taxa and diversity indices for benthic macroinvertebrates in the estuaries are shown in Fig. 5. Kakum, Volta and Ankobra Estuaries respectively recorded 822, 519 and 164 specimens during the sampling period with Kakum (18) and Volta (17) having a relatively high number of taxa compared to Ankobra Estuary (7). The highest and lowest diversity was recorded in Kakum and Ankobra Estuary, respectively. Additionally, relatively high species evenness and species richness was found in Kakum Estuary in comparison to the Volta and Ankobra Estuaries (see details Fig. 5).

### 3.5 Species-environment interactions

**Fig. 6:**
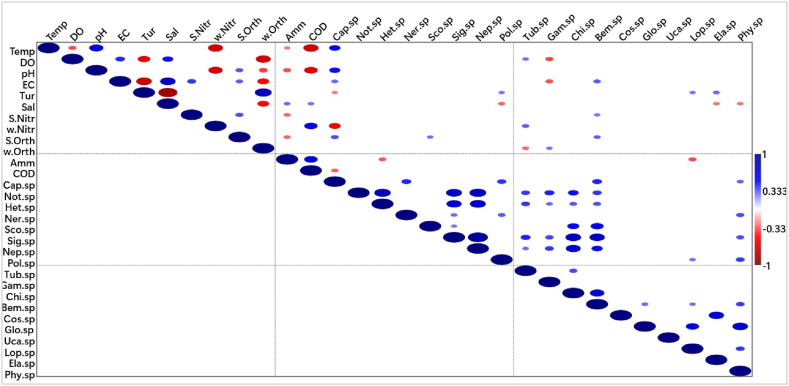
Spearman's rank order correlation for Kakum Estuary. **Note**: In the correlation matrices, deep blue and deep red colour indicate positive and negative correlations, respectively. The deeper the colour the stronger the correlation. The circles indicate p < 0.05 and the larger the size of the circle the stronger the correlation ***Abbreviations****: Temp-temperature, DO-dissolved oxygen, EC- electrical conductivity, COD-chemical oxygen demand, Turb-turbidity, Sal-salinity, w-Nitr-nitrogen-nitrogen in water, S-Nitr-nitrogen-nitrogen in sediment, w-Orth-orthophosphate in water, S-Orth-orthophosphate in sediment, Amm-ammonium-nitrogen, Cap-Capitella, Not-Notomastus, Het-Heteromastus, Ner-Nereis, Sco-Scoloplos, Sig-Sigambra, Neph-Nephtys, Poly-Polyphysia, Tubi-tubifex, Gam-Gammarus, Chir -Chironomous, Bem -Bemlos, Cos-Cossura, Glossi -Glossiphonia, Lop- Lopadorrhynchus, Elas-Elasmopus, Phyll-Phyllodoce*. (For interpretation of the references to colour in this figure legend, the reader is referred to the Web version of this article.)

Associations were observed among individual physicochemical parameters and different benthic fauna using the Spearman's Rank Order Correlation. In the Kakum Estuary, a number of associations were observed among individual physicochemical parameters and among the different benthic fauna (Fig. 6).

Furthermore, more associations in the interest of the present study were documented between physicochemical parameters and benthic macroinvertebrates. For instance, a weak positive but significant correlation (r = 0.284) was observed between DO and *Tubifex* sp. Also, significant moderate correlations existed between EC and *Capitella* sp. (r = 0.307) as well as EC and *Bemlos* sp. (r = 0.332). Turbidity and COD were weakly (r = −0.270) and moderately (r = - 0.323) correlated with *Capitella* sp. The two correlations were both negative but significant at p = 0.05.

In the same Kakum Estuary, turbidity correlated weakly but positively with both *Polyphysi*a sp. (r = 0.274) and *Lopadorrhynchus* sp. (r = 0.276). However, it showed a moderate but positive correlation with *Elasmopus* sp. (r = 0.302). All the correlations between benthic fauna and turbidity were significant at p = 0.05. Just like turbidity, salinity displayed significant associations with benthic fauna at p = 0.05. While salinity and *Polyphysia* sp. Correlated moderately and negatively (r = −0.315), weak negative associations were observed between salinity and both *Elasmopus* sp. and *Phyllodoce* sp. (r = −0.29). Moreover, nutrients correlated significantly with benthic fauna at p = 0.05, with nitrates and orthophosphates demonstrating positive correlations while negative correlations were observed in ammonium-nitrogen. *Bemlos* sp. was weakly and moderately correlated with nitrate-nitrogen (r = 0.275) and orthophosphates (r = 0.315), respectively. In addition to that, weak positive correlations were recorded between orthophosphates and *Scoloplos*. sp (r = 0.285) as moderate negative associations existed between ammonium-nitrogen and *Heteromastus* sp. (r = −0.338).

**Fig. 7:**
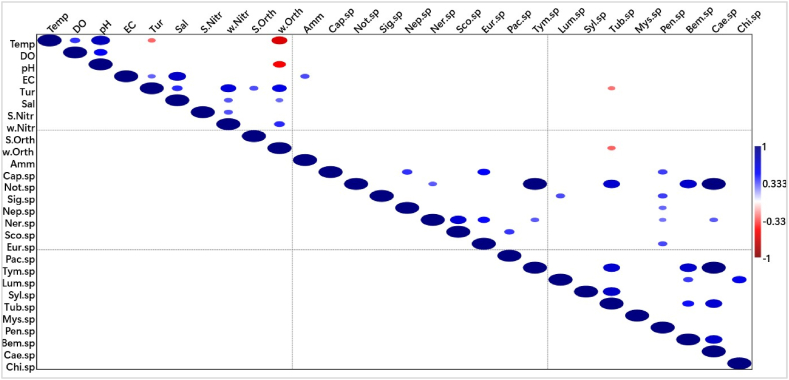
Spearman’ rank order correlation for Volta Estuary. **Note:** In the correlation matrices, deep blue and deep red colour indicate positive and negative correlations, respectively. The deeper the colour the stronger the correlation. The circles indicate p < 0.05 and the larger the size of the circle the stronger the correlation **Abbreviations**: Temp-temperature, DO-dissolved oxygen, EC- electrical conductivity, COD-chemical oxygen demand, Turb-turbidity, Sal-salinity, w-Nitr-nitrogen-nitrogen in water, *S*-Nitr-nitrogen-nitrogen in sediment, w-Orth-orthophosphate in water, *S*-Orth-orthophosphate in sediment, Amm-ammonium-nitrogen, Cap-Capitella, Not-Notomastus, Sig-Sigambra, Neph-Nephtys, Ner-Nereis, Sco-Scoloplos, Eur-Euridice, Pach-Pachymelania, Lumbr-Lumbriconereis, Syll-Syllis, Tubi-Tubifex, Mys-Mysis, Pen-Penaeus, Bem -Bemlos, Caen-Caenobita, Chiron -Chironomid. (For interpretation of the references to colour in this figure legend, the reader is referred to the Web version of this article.)

In the Volta Estuary, the only correlation between physicochemical parameters and benthic macroinvertebrates existed between turbidity and *Tubifex* sp. as well as *Tubifex* sp. and orthophosphates in the water column (r = −0.312). Although the correlations were negative and weak (r = −0.278), they were significant at p = 0.05. In Fig. 7, the other associations were among individual physicochemical parameters as well as among individual macroinvertebrate fauna.

**Fig. 8:**
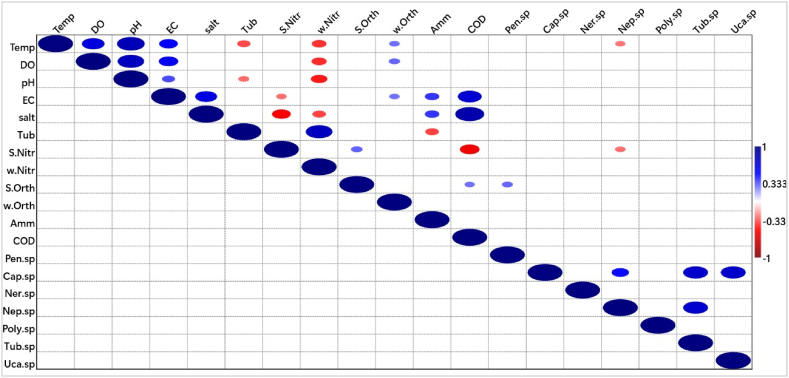
Spearman’ rank order correlations for Ankobra Estuary. **Note**: In the correlation matrices, deep blue and deep red colour indicate positive and negative correlations, respectively. The deeper the colour the stronger the correlation. The circles indicate p < 0.05 and the larger the size of the circle the stronger the correlation **Abbreviations**: Temp-temperature, DO-dissolved oxygen, EC- electrical conductivity, Turb-turbidity, Sal-salinity, w-Nitr-nitrogen-nitrogen in water, *S*-Nitr-nitrogen-nitrogen in sediment, w-Orth-orthophosphate in water, *S*-Orth-orthophosphate in sediment, Amm-ammonium-nitrogen, COD-chemical oxygen demand, Pen-Penaeus, Cap-Capitella, Ner-Nereis, Neph-Nephtys, Poly-Polyphysia, Tubi-Tubifex. (For interpretation of the references to colour in this figure legend, the reader is referred to the Web version of this article.)

In the Ankobra Estuary, salinity depicted a negative correlation with *Penaeus* sp*.* as seen in Fig. 8. However, the correlation was weak (r = −0.293) whereas no other significant associations existed between physicochemical parameters and faunal specimen in Ankobra Estuary.

## 4 Discussion

### 4.1 Physicochemical parameters

The most restricting environmental elements in aquatic ecosystems include physicochemical parameters such as temperature, DO, EC, pH, and turbidity [60]. The distribution of organisms, as well as other processes regulating metabolism and other changes in waterbodies, are all significantly influenced by SWT [61]. The relatively low SWT in Kakum Estuary (27.9°C) could be due to shading from mangrove trees observed at either side of the estuarine banks since the estuary has been listed as the most diverse mangrove forest in Ghana [64]. The SWT in the current study is within the range of values reported in other studies within Ghana. For instance, similar findings of SWT by authors like Adjei-Boateng et al. [65] and Madkour et al. [66] in the Volta Estuary, Dzakpasu et al. [33] and Fianko et al. [55] in the Kakum Estuary, and Faseyi et al. [37] and Soetan et al. [67] in the Ankobra Estuary corroborate the findings in the current study.

The minimum and maximum DO concentrations were recorded in Ankobra (5.4 mg/L) and Volta Estuaries (6.1 mg/L), respectively. The relatively minimum DO in Ankobra Estuary could be attributed to high siltation from gold mining activities in the catchment area, which affected sunlight penetration hence reducing photosynthetic activities that consequently affected DO supply. On the other hand, the DO levels in the Volta Estuary were notably higher than those in the Ankobra Estuary due to the limited amount of silt and suspended matter that reaches the estuary, which is a result of the river being dammed at Akosombo. This results in limited biodegradation and organic decomposition activity. Additionally, there are fewer impacts, especially from gold mining, which is much less common in the Volta Estuary compared to the Ankobra Estuary. Similar findings have been reported in the literature, for instance, Dzakpasu et al. [33] in Kakum Estuary and those of Adjei-Boateng et al. [65] and Madkour et al. [66] in the Volta Estuary fall within the ranges (Table 2) of the findings in the current study. Contrary to this, wider DO ranges were recorded in Pra Estuary [68] and some coastal waters in Nigeria [69]. Concentrations of DO under 5 mg/L may have a major impact on the survival and normal functioning of biological communities while DO levels below 2 mg/L may result in hypoxic conditions [70]. According to the Ghana Raw Water Criteria and Guidelines, the Target Water Quality Range (TWQR) of DO for sustaining aquatic life is 5.0–8.0 [71]. The DO values from the current study therefore are suitable for aquatic life in this circumstance.

Electrical conductivity refers to the capacity of water to conduct electric current based on the amount of ionised compounds present in it [72]. According to the TWQR of the Ghana Raw Water Quality Criteria and Guidelines, any water with an EC of 0 μS/cm to 70 μS/cm [73] is best for preserving the well-being of aquatic ecosystems. Results from the current study indicated that the median EC values recorded in the three estuaries were significantly above the maximum limit of TWQR range. Also, the reports from other studies in similar environments that corroborate the current study include Aheto et al. [74] in Whin Estuary, Okyere et al. [75] in Kakum Estuary and Faseyi et al. [76] in Ankobra and Pra Estuaries. Nevertheless, contrary to the current study, low EC values were reported by Adjei-Boateng et al. [65] and Madkour et al. [66] in the Volta Estuary. The findings of this study suggest that the three estuaries have significant concentrations of dissolved ions, including inorganic salt and organic litter which may be due to domestic wastewater discharges and surface runoffs from cultivated fields in the catchment area.

When combined with other environmental factors, the pH of water can have a significant impact on aquatic life due to the acidity or alkalinity of waterbodies. According to the National Estuarine Research Reserve, NERR [77], aquatic life is said to be most adaptive in a pH range between 5 and 9. The acidic or alkaline state of estuaries could be influenced by the amount of alkaline ions in seawater and the bedrock of the waterbody [33]. The present study recorded wider pH ranges, suggesting a potential buffering impact of seawater. Similar observations have been made in Tapi Estuary, India [78]. In a different view, other studies have reported narrow pH ranges including Tufuor et al. [38,79] in the Pra Estuary, Adjei-Boateng et al. [65] and Madkour et al. [66] in the Volta Estuary and Dzakpasu et al. [33] in Kakum Estuary. The results of this study indicate that the estuaries can support aquatic life.

High turbidity in water could result from siltation, watershed runoffs, aquatic weeds and other organic compounds produced by dead and decayed plant matter which gives waterbodies a rust-red colouration [80]. In this study, the maximum turbidity levels were above 3000 NTU in the Ankobra Estuary and as low as 0.0 NTU in the Volta Estuary. The turbidity levels above 3000 NTU, which is of concern in the Ankobra Estuary may be due to significant amounts of silt inflow from upstream regions where illegal small scale gold mining activities occur. Consequently, high turbidity increases the heat absorption capacity of water, leading to higher temperatures that subsequently lower the concentration of oxygen and ultimately affect primary productivity. Also, it impairs biological activities by decreasing disease resistance and clogging fish gills [38]. High turbidity also induces cloudiness in the water and decreases visibility, which hinders breeding, feeding, reproduction, and ultimately the survival of aquatic life [81,82]. The turbidity in the current study was found to be comparably higher than what was recorded in earlier works in the same estuary [38,67]. This pattern indicates increasing impacts of siltation with time as a result of illegal mining activities. Likewise, the results of the current study support those from the Pra Estuary, where turbidity levels above 1000 10.13039/100010016NTU have been reported [82]. According to Bilotta and Brazier [81], the maximum turbidity for aquatic life is 100 NTU, whereas turbidity levels over 500 NTU are considered harmful to aquatic life [82]. With the exception of Ankobra Estuary, the turbidity of Kakum and Volta Estuaries is suitable for supporting aquatic life.

Although high nutrient concentrations in estuaries increase primary productivity, high turbidity levels in estuaries make it difficult for light to penetrate the water column. During periods of intense rainfall, significant volumes of deposited nitrates in soils from industrial and agricultural operations are carried into estuaries, and this results in an increase in turbidity [72,83]. In the current study, the concentration of NO_3_-N in the water column was lower than in sediments in the three estuaries. This could be beacuse sediments offer a larger surface area and porous environment that allows for the retention of nitrates. Particularly, nitrates can bind to sediment particles, making it less prone to rapid removal through processes like denitrification or assimilation by organisms in the water column [84]. In Kakum and Ankobra Estuaries, slightly higher values of NO_3_–N than the recommended limit of 1.0 mg/L for NO_3_–N in estuaries and coastal ecosystems to prevent algal blooms was recorded [85]. A similar finding of high NO_3_–N concentrations has been reported in Pra Estuary [79]. On the other hand, lower concentrations below 2 mg/L were reported from similar waterbodies by other studies, including Faseyi et al. [51] in the Pra and Ankobra Estuaries, and CRC/FoN [86] in the Ankobra Estuary.

Moreover, the concentration of orthophosphates was generally higher in sediment matrix than in the water column in Kakum and Volta Estuaries. Sediments can host specific microbial populations that contribute to phosphorus transformations, such as phosphorus-solubilising bacteria and phosphorus-accumulating bacteria. These microbes can alter the balance of phosphorus species in sediments, leading to higher concentrations relative to the water column [87]. Furthermore, the average orthophosphate concentration in the threeestuaries was above the recommended 0.1 mg/L for estuaries and other coastal ecosystems [85]. These results contradict what Tufuor et al. [79] and Faseyi et al. [38] reported in the Pra Estuary as well as reports from the collaborative report between Coastal Research Centre and Friends of the Nation-Ghana [88] in the Ankobra Estuary where lower values were recorded. The variation in the concentrations of both nitrate-nitrogen and orthophosphates in the same waterbodies could be due to run-offs from phosphorus-rich sewage, agricultural run-offs, and high levels of organic matter decomposition from the surrounding mangrove ecosystems as possible sources of phosphorus in the estuaries. In comparison to NO_3_–N and ${\mathrm{PO}}_{4}^{3-}$, NH_4_–N levels were relatively high in all estuaries (≥39 mg/L) . In areas of low DO concentration, NO_3_–N is easily transformed to NH_4_–N and this could be true for the current study since relatively low DO values (≤6.1 mg/L) were recorded in the estuaries.Moreover, elevated levels of NH_4_-N in waterbodies point to potential presence of contaminants from human or animal waste, hence a danger to human and animal health. Also, in elevated concentrations is toxic to aquatic lfe. It poses a challenge to be efficiently excreted [89]

### 4.2 Occurrence and composition of benthic macroinvertebrates

Annelids, crustaceans and molluscs are the three most common macroinvertebrates found in estuaries [90]. It has been demonstrated that these groups accurately represent a variety of aquatic environments in West Africa, including estuaries and lagoons [91]. In the current study, 26 taxa belonging to 24 families and six classes were encountered, summing up to 1505 specimens collected during the sampling period. Individually, the three estuaries recorded 18, 17 and 7 taxa in Kakum, Volta and Ankobra Estuaries, respectively. The number of taxa identified in Kakum and Volta was comparable to the 17 taxa reported in the Gambia River Estuary [91]. On the contrary, the number of taxa recorded in each of the three estuaries was comparatively lower than the 87 taxa reported in Brazilian tropical Estuaries [92].

Kakum and Volta Estuaries were dominated by polychaetes, which is similar to observations made by Adam et al. [91]. Taxa such as *Capitella* sp., *Nereis* sp. and *Nephtys* sp. were found to be ubiquitous in the three estuaries. Pollution-tolerant species like *Capitella* sp. have been reported to tolerate low oxygen conditions, hence considered an indicator of organic pollution [93,94]. Additionally, *Nereis* sp. and *Nephtys* sp. have been observed to tolerate wide ranges of salinity [95] and according to Balogun et al. [90], *Nereis* sp. can indicate pollution in an aquatic environment. *Cossura* sp., with one specimen recorded in Kakum Estuary, has been designated as pollution tolerant and its presence or absence can be used to determine ecosystem health [93,96]. While *Chironomous* sp. can tolerate high levels of pollution and low DO [31,97,98], *Tubifex* sp. can withstand severely enriched environments with organic pollution [27,97]. The dominance, abundance and presence of pollution-tolerant taxa like *Capitella* sp., *Nereis* sp., *Heteromastus* sp., *Tubifex* sp., *Cossura* sp. and *Chironomous* sp. in Kakum Estuary suggest an organically polluted environment. Other studies have demonstrated the presence of pollution indicator species in brackish water in West Africa [31,33,34,91].

*Scoloplos* sp. was encountered in low abundance in Kakum Estuary (four specimens), relatively high abundance in the Volta Estuary (63 specimens) and no occurrence in Ankobra Estuary. This particular taxon has been said to be sensitive to pollution [99]. This could be indicative of a conducive ecosystem health in the Volta Estuary in comparison to the other estuaries where the conditions are not conducive for survival of the organism to thrive. Additionally, *Gammarus* sp., which was particularly recorded in Kakum Estuary in low abundance (26 specimens) and zero occurence in Volta and Kakum Estuaries has been observed to dominate less polluted waters hence relatively sensitive to pollution [100]. The low abundance of *Gammarus* sp. in Kakum Estuary and zero occurrence in the other estuaries and could point to some level of pollution in the estuaries. In the Volta Estuary, *Tympanotonus* sp. and *Pachymelania* sp. (gastropods) were documented and seemed to be endemic to the estuary. Although *Tympanotonus* sp. has been reported to be pollution tolerant [101,102]. *Pachymelania* sp. is sensitive to environmental changes including the presence of contaminants in the water [90,101]. The occurrence of *Pachymelania* sp .in the Volta Estuary could be a result of the existence of food resources such as detritus for their survival, favourable physicochemical conditions (i.e., low turbidity, high DO, and relatively higher SWT), and less predation pressure. Consequently, the absence of *Pachymelania* sp. in Kakum and Ankobra Estuaries is indicative of contamination in these estuaries.

Additionally, some polychaetes in the Lumbrineridae family are negative indictors of poor benthic conditions and their absence in a community is an indicator of poor environmental conditions [93]. With eight specimens of *Lumbriconereis* sp. documented in the Volta Estuary and zero occurrence in Kakum and Ankobra Estuaries, it is an indication that all the estuaries have poor environmental conditions, except Volta with relatively good conditions. The presence of pollution sensitive taxa like *Scoloplos* sp., *Eurydice* sp., *Lumbriconereis* sp. and *Pachymelania* sp. in the Volta Estuary could be an indication of a relatively less polluted environment. The high abundance of *Penaeus* sp. in Ankobra Estuary could be explained by their ability to tolerate wider salinity ranges of whereas other organisms could not [103] hence could be used as an indicator of salinity. The low abundance of benthic macroinvertebrates in Ankobra Estuary in general is attributed to elevated turbidity levels that increases siltation. On the contrary, in the Volta Estuary, the water is free from turbidity and siltation, attracting largely pollution sensitive organisms.

### 4.3 Diversity of benthic macroinvertebrates

Environmental factors and stressors in a waterbody have a significant impact on the number and diversity of benthic macrofauna [104]. The structure, distribution, diversity, and composition of the benthic macroinvertebrate communities are also influenced by habitat physiographic features and microhabitat variety [31]. In the current study, Ankobra Estuary recorded the highest turbidity resulting from sedimentation of the estuary from mining activities in the catchment and had only 164 macroinvertebrate specimens distributed among seven taxa in the entire hydrological year; recorded the lowest species diversity, richness, and evenness. This scenario could be linked to negative impacts of turbidity including blockage of gills of filter-feeding benthic fauna, and reduction of visibility that consequently affects other physiological processes that eventually disrupt the survival of benthic fauna [82]. Although oligochaetes have been found to survive silted environments [105], only one specimen was identified in the estuary. Furthermore, low species diversity in the Ankobra Estuary could be due to low DO concentration that implied disruption of reproductive cycles and food chain [106].

On the other hand, relatively high macroinvertebrate diversity, richness and evenness was recorded in Kakum Estuary. These findings are comparable to other studies in similar environments including Pra Estuary [34] and Whin Estuary [86]. Moreover, the findings in the Volta and Ankobra Estuaries where species diversity (H’<2) has also been documented in the Gambia River Estuary [91]. Similarly, high species richness (d = 17.85) and evenness (J = 0.79) were recorded in Kakum Estuary. These values are comparatively higher than the maximum values obtained for species richness and evenness in Pra Estuary [34].

Species diversity largely depends on species richness, abundance and distribution in an ecosystem. In the current study, Kakum Estuary, which had the highest species evenness and richness, recorded the highest species diversity (H’ = 2.28) while the Ankobra, with lowest species evenness and richness, had the lowest species diversity (H’ = 0.33). In general, high diversity index values above 3 (H’>3) point to a stable, balanced habitat, while values below one (H’<1) are indicative of a polluted and degraded habitat [107]. Pielou's evenness index ranges from 0 to 1; closer to 1 means a more even distribution [108]. Since Margalef's index has no upper bound, it is used for spatial comparison [109]. The fact that diversity (H'≤3), evenness (J' ≤0.8), and richness (d ≤18) for the three estuaries is not necessarily indicative of contamination instability and imbalance. Estuaries are very dynamic ecosystems, and very few taxa are able to adapt to the frequently changing environmental conditions and cope with the fluctuating environmental stress. Species diversity is a prime aspect of biological monitoring and is considered a valuable indicator in determining ecosystem health [110]. Therefore, ranking the three estuaries in terms of stability using biological indices implies that Kakum Estuary is ecologically healthier than Volta Estuary, which is in turn healthier than Ankobra Estuary, ie., Estuarine ecological health ranking; Kakum > Volta ˃ Ankobra Estuary.

### 4.4 Species-environment interactions

Physicochemical factors can have a favourable or detrimental impact on the entire benthic population in any aquatic habitat depending on their levels or concentrations. Therefore, the richness, abundance and composition of benthic communities can change over time due to changes in physicochemical factors. A diverse population of benthic fauna shows that the water quality supports their existence in the entire ecosystem. The abundance and diversity of macroinvertebrates in aquatic environments have been linked to some parameters such as temperature, salinity, DO, nutrient concentrations, pH, turbidity, and organic matter [111].

Salinity is one of the key determinants of macroinvertebrate diversity and abundance in Ghanaian coastal waters and benthic macroinvertebrate communities react differently to variations in water salinity [30]. In the Kakum Estuary, some polychaetes like *Polyphysia* sp. and *Phyllodoce* sp. demonstrated moderate and weak negative correlations, respectively with salinity. Furthermore, some crustaceans like *Elasmopus* sp. and *Penaeus* sp. showed significant but weak negative correlations with salinity in Kakum and Ankobra Estuaries, respectively. The findings are contradictory to the fact that polychaetes as deposit feeders increase in abundance with salinity, but agree with observations that suspension feeders like crustaceans decrease in abundance with salinity [112]. Tachet et al. [113] points out that lower salinity levels favour the development of sensitive species like *Penaeus* sp. Given its euryhaline nature [103] and high percentage composition (94 %) in Ankobra Estuary, the taxon can be considered an indicator taxon of salinity.

The concentrations of DO that support aquatic life range from 5.0 mg/L to 8.0 mg/L [71]. In aquatic life settings where DO concentrations range from 3.5 mg/L to 6.5 mg/L, a greater macroinvertebrate fauna is favoured [34]. However, some benthic faunae can thrive well in heavily organic-contaminated environments (i.e., organisms in the families of Tubificidae, Capitellidae and Cirratulidae) indicating stressed communities [93]. In the present study, *Tubifex* sp. (oligochaete) demonstrated a weak but significant positive correlation with DO in the Kakum Estuary. This finding disagrees with Ertaş and Yorulmaz [114] and Barrilli et al. [27] who observed a negative association between *Tubifex* sp. and DO. Another study has indicated that *Tubifex* sp. can reach very high densities in organic-polluted systems [34] as they feed on organic matter from oxygen-poor sediments [27]. The present findings in Kakum Estuary where only nine specimens were encountered could be indicative that the estuary is moderately polluted with organic pollutants.

Electrical conductivity has been recognised as the ability of water to conduct electric current and as an indicator of both dissolved ions and salts in water [72]. In the present study, EC positively and significantly showed a moderate correlation with *Capitella* sp. In the Kakum Estuary, increased EC in the water (7580 µS/cm) could have been as a result of high surface run-off of organic debris from anthropogenic sources like domestic sewage since the Kakum river drains a densely populated and highly urbanised Cape Coast in the Central Region of Ghana. The findings concur with other studies where EC positively and moderately correlated with *Capitella* sp. in coastal waters [115,116].

Effects of turbidity on benthic macroinvertebrates have been demonstrated [38,82]. This is probably the reason for the negative correlation between turbidity and *Capitella* sp. despite their ability to colonise organically enriched environments. Additionally, the positive correlations observed between turbidity and some polychaete taxa like *Polyphysi*a sp. in Kakum Estuary is because, *Polyphysia* sp. is associated with high turbidity areas, which are in turn associated with abundant food resources like detritus and plankton. Some other *Polyphysia* sp. like *Scalibregma crassa*, are known to thrive in areas with moderate to high turbidity, indicating tolerance to high conditions [34]. In the Volta Estuary, the negative and weak correlations between turbidity and *Tubifex* sp. could be explained by very low turbidity values recorded (sometimes 0.0 NTU) which were not favourable for the thriving of this particular taxon. Also, only three specimens of *Tubifex* sp. were recorded in the Volta Estuary, which explains the negative correlation with turbidity.

High concentrations of nutrients in water are a characteristic of diffuse sources of organic and inorganic matter from anthropogenic activities in the catchment area [116]. However, the influence of nutrients on benthic fauna in Kakum Estuary was less impactful as seen from the weak positive correlations between some taxa like *Bemlos* sp. and *Scoloplos* sp. (pollution-sensitive taxa) with NO_3_–N and ${\mathrm{PO}}_{4}^{3-}$. Also, the weak associations could be related to the very low levels of NO_3_–N and ${\mathrm{PO}}_{4}^{3-}$ recorded in the estuary. Additionally, the negative association between NH_4_–N and *Heteromastus* sp. (pollution-tolerant taxon) could imply that the occurrence of this particular taxa in the estuary is not entirely influenced by the high NH_4_–N concentration but by other factors. The study by Ertaş and Yorulmaz [114] contradicts the present study by ascertaining that the distribution of pollution-tolerant taxa positively correlated with NO_3_–N, ${\mathrm{PO}}_{4}^{3-}$ and NH_4_–N. The findings of the current study show that Kakum Estuary is low on nutrient enrichment and the pollution-tolerant taxa encountered there are not fully influenced by nutrient availability.

The study gives an overview of the spatial distribution of benthic macroinvertebrates in three estuaries along the coast of Ghana and the use of benthic communities as indicators of the estuarine conditions. Pollution tolerant taxa (*Capitella* sp., *Nereis* sp., *Heteromastus* sp., *Tubifex* sp., *Cossura* sp. and *Chironomous* sp.), pollution sensitive taxa (*Scoloplos* sp., *Eurydice* sp., *Lumbriconereis* sp. and *Pachymelania* sp.) and salinity indicator taxon (*Penaeus* sp.) were recorded in Kakum, Volta and Ankobra Estuaries, respectively. Although Kakum Estuary is dominated by a wide range of pollution-tolerant taxa, Spearman rank order correlation analysis shows weak and moderate correlations, suggesting that the estuary is in moderate condition based on organic pollution. Moreover, Kakum is the most diverse estuary despite the moderate pollution levels. However, in the three estuaries, the quality of water is a factor of anthropogenic activities in the catchment area which has negative implications as expressed through physicochemical parameters like DO, turbidity, nutrients, EC and COD. As seen from literature, estuarine ecosystem health has been indicated using physicochemical and benthic macroinvertebrate data in isolation. The study recommends integration of the various water quality metrics into a model that will provide a holistic view of estuarine ecosystem health, including frequent monitoring of water quality.

## 5 Conclusion

The current study has disclosed significant acumens into the presence and extent of pollution in estuaries along the coast of Ghana. The dominance of pollution-sensitive, pollution-tolerant and salinity indicator benthic fauna in the selected estuaries, in conjunction with their associations with crucial environmental factors indicate variation in levels of pollution. In terms of ecological health status, Ankobra Estuary emerged as the least healthy, followed by Volta Estuary, then the Whin Estuary and finally the Kakum Estuary being the healthiest. These results highlight the need to protect estuarine ecosystem health from further degradation with anthropogenic sources of contaminants, especially the Ankobra and Kakum Estuaries. The findings emphasise the need to integrate data obtained from benthic macroinvertebrates and environmental parameters that indicate the status of water quality into the development of a water quality monitoring model for easy assessment of the estuarine ecosystem health in Ghana.

## Funding

This research work was funded by the Africa Centre of Excellence in Coastal Resilience (ACECoR), University of Cape Coast, with support from the 10.13039/100004421World Bank and the Government of Ghana, 10.13039/100004421World Bank 10.13039/100005362ACE Grant Number is credit number 6389-G. Dorothy Khasisi Lukhabi was supported by a small research grant (Tonolli Award) by the International Society of Limnology (10.13039/100001357SIL).

## Data availability

Data will be made available on request.

## CRediT authorship contribution statement

**Dorothy Khasisi Lukhabi:** Conceptualization, Data curation, Formal analysis, Funding acquisition, Investigation, Methodology, Visualization, Writing – original draft, Writing – review & editing. **Paul Kojo Mensah:** Supervision, Writing – review & editing. **Noble Kwame Asare:** Supervision, Writing – review & editing. **Margaret Fafa Awushie Akwetey:** Formal analysis, Writing – review & editing. **Charles Abimbola Faseyi:** Methodology, Visualization, Writing – review & editing.

## Declaration of competing interest

The authors declare that they have no known competing financial interests or personal relationships that could have appeared to influence the work reported in this paper.

## References

[1] Potter I.C., Chuwen B.M., Hoeksema S.D., Elliott M. (2010). The concept of an estuary: a definition that incorporates systems which can become closed to the ocean and hypersaline. Estuar. Coast Shelf Sci..

[2] Ujjania N.C., Dubey M. (2015). Water quality index of estuarine environment. Curr. Sci..

[3] Barbier E.B., Hacker S.D., Kennedy C., Koch E.W., Stier A.C., Silliman B.R. (2011). The value of estuarine and coastal ecosystem services. Ecol. Monogr..

[4] Meire P., Ysebaert T., Van Damme S., Van Den Bergh E., Maris T., Struyf E. (2005). The Scheldt estuary: a description of a changing ecosystem. Hydrobiologia.

[5] Day J.W., Yáñez‐Arancibia A., Kemp W.M., Crump B.C. (2012). Introduction to estuarine ecology. Estuar. Ecol..

[6] Thrush S.F., Dymond J. (2013). Ecosystem Services in New Zealand: Conditions and Trends.

[7] Ducrotoy J.-P. (2019). Coasts and Estuaries.

[8] Van Niekerk L. (2013). Country-wide assessment of estuary health: an approach for integrating pressures and ecosystem response in a data limited environment. Estuar. Coast Shelf Sci..

[9] Wiethüchter A., Assessment of ecosystem goods and services provided by the coastal zone system limfjord, DTU Aqua, Danmarks Tekniske Universitet (2008). Report no. 192-08, http://www.aqua.dtu.dk/Publikationer/Forskningsrapporter/Forskningsrapporter_siden_2008.

[10] Vugteveen P., Leuven R.S.E.W., Huijbregts M.A.J., Lenders H.J.R. (2006).

[11] Borja A. (2013). Good environmental status of marine ecosystems: what is it and how do we know when we have attained it?. Mar. Pollut. Bull..

[12] Lu Y. (2015). Ecosystem health towards sustainability. Ecosys. Health Sustain..

[13] Harwell M.A. (2019). Conceptual framework for assessing ecosystem health. Integrated Environ. Assess. Manag..

[14] Oertel J., N, Salánki, Ambasht RS A.N. (2003). Modern Trends in Applied Aquatic Ecology.

[15] Parmar T.K., Rawtani D., Agrawal Y.K. (2016). Bioindicators: the natural indicator of environmental pollution. Front. Life Sci..

[16] Tampo L., Kaboré I., Alhassan E.H., Ouéda A., Bawa L.M., Djaneye-Boundjou G. (2021). Benthic macroinvertebrates as ecological indicators: their sensitivity to the water quality and human disturbances in a tropical river. Front. Water.

[17] Lavoie S., I, Campeau (2010). Fishing for diatoms: fish gut analysis reveals water quality changes over a 75-year period. J. Paleolimnol..

[18] Gresens S., Smith R., Sutton-Grier A., Kenney M. (2010). Benthic macroinvertebrates as indicators of water quality: the intersection of science and policy. Terr. Arthropod Rev..

[19] Reynoldson T.B., Metcalfe-Smith J.L. (1992). An overview of the assessment of aquatic ecosystem health using benthic invertebrates. J. Aquat. Ecosys. Health.

[20] Bressler D.W., Stribling J.B., Paul M.J., Hicks M.B. (2006). Stressor tolerance values for benthic macroinvertebrates in Mississippi. Hydrobiologia.

[21] Nerbonne B.A., Vondracek B. (2001). Effects of local land use on physical habitat, benthic macroinvertebrates, and fish in the Whitewater River, Minnesota, USA. Environ. Manag..

[22] Karmakar P., Pal S., Mishra M. (2022). Arthropods: an important bio-indicator to decipher the health of the water of south Asian rivers,” *River Heal. Ecol. South Asia Pollution*. Restoration, Conserv..

[23] Nunkumar S., Monitoring the health of the rivers of the Durban Metropolitan Area using fresh water invertebrates, A pilot study (2002). Unpublished MSc thesis of the University of DurbanWestville. Durban.

[24] Van Dolah R.F., Hyland J.L., Holland A.F., Rosen J.S., Snoots T.R. (1999). A benthic index of biological integrity for assessing habitat quality in estuaries of the southeastern USA. Mar. Environ. Res..

[25] Ferraro S.P., Swartz R.C., Cole F.A., Schults D.W. (1991). Temporal changes in the benthos along a pollution gradient: discriminating the effect of natural phenomena from sewage-industrial wastewater effects. Estuar. Coast Shelf Sci..

[26] Pinto R., Patrício J., Baeta A., Fath B.D., Neto J.M., Marques J.C. (2009). Review and evaluation of estuarine biotic indices to assess benthic condition. Ecol. Indicat..

[27] Barrilli G.H.C., Negreiros N.F., Rocha O., Verani J.R. (2021). Macroinvertebrates responses based on chemical and physical variables in urban streams. Pap. Avulsos Zool. (Sao Paulo).

[28] Zhang Y. (2019). Nutrient enrichment homogenizes taxonomic and functional diversity of benthic macroinvertebrate assemblages in shallow lakes. Limnol. Oceanogr..

[29] Gordon C. (2000). Hypersaline lagoons as conservation habitats: macro_invertebrates at Muni Lagoon, Ghana. Biodivers. Conserv..

[30] Lamptey E., Armah A.K. (2008). Factors affecting macrobenthic fauna in a tropical hypersaline coastal lagoon in Ghana, West Africa. Estuar. Coast.

[31] Aggrey-Fynn J., Galyuon I., Aheto D.W., Okyere I. (2011). Assessment of the environmental conditions and benthic macroinvertebrate communities in two coastal lagoons in Ghana. An. Biol. Reserach.

[32] Armah F.A., Ason B., Luginaah I., Essandoh P.K. (2012). Characterization of macro-benthic fauna for ecological health status of the Fosu and Benya lagoons in coastal Ghana. J. Ecol. F. Biol..

[33] Dzakpasu M.F.A., Yankson K., Jnr J.B. (2015). Comparative study of the benthic macroinvertebrate communities of two estuaries on the Southwestern Coast of Ghana. Ann. Biol. Res..

[34] Okyere I., Nortey D.D.N. (2018). Assessment of the ecological health status of river pra estuary (Ghana) and adjoining wetland using physico-chemical conditions and macroinvertebrate bioindicators. West African J. Appl. Ecol..

[35] Margaret Fafa Awushie Dzakpasu (2019).

[36] Faseyi C.A., Miyittah M.K., Yafetto L. (2023). Assessment of environmental degradation in two coastal communities of Ghana using Driver Pressure State Impact Response (DPSIR) framework. J. Environ. Manag..

[37] Faseyi C.A., Miyittah M.K., Yafetto L., Sowunmi A.A., Lutterodt G. (2022). Heliyon Pollution Fingerprinting of Two Southwestern Estuaries in Ghana.

[38] C. A. Faseyi, M. K. Miyittah, A. A. Sowunmi, and L. Yafetto, “Water quality and health risk assessments of illegal gold mining-impacted estuaries in Ghana,” Mar. Pollut. Bull., vol. 185, no. PA, p. 114277, doi: 10.1016/j.marpolbul.2022.114277.

[39] Klubi E., Abril J.M., Nyarko E., Delgado A. (2018). Impact of gold-mining activity on trace elements enrichment in the West African estuaries: the case of Pra and Ankobra rivers with the Volta estuary (Ghana) as the reference. J. Geochem. Explor..

[40] Nortey D.D.N., Aheto D.W., Blay J., Jonah F.E., Asare N.K. (2016). Comparative assessment of mangrove biomass and fish assemblages in an urban and rural mangrove wetlands in Ghana. Wetlands.

[41] Okyere E.Y., Adu-Boahen K., Boateng I., Dadson I.Y., Boanu N.Y., Kyeremeh S. (2023). Analysis of ecological health status of the Muni Lagoon: evidence from heavy metal content in its water and fish samples. Geo Geogr. Environ..

[42] Ansa-Asare O., Mensah E., Entsua-Mensah M., Biney C. (2009). Impact of human activities on nutrient and trophic status of some selected Lagoons in Ghana. West African J. Appl. Ecol..

[43] Chuku E.O., Yankson K., Obodai E.A., Acheampong E., Aheto D.W. (2023).

[44] Agblemanyo F.E. (2021). Ghana and Hydrographic Conditions of Their Habitats.

[45] Sowah S.A. (2019).

[46] Adu-Boahen K., Boateng I., Okyere E.Y., Kyeremeh S. (2023). An assessment of water quality and the locals’ perception of coastal lagoon pollution in Ghana: A case study of Chemu lagoon in Tema. Asian Rev. Environ. Earth Sci..

[47] Vowotor S.M., M K., Odumah Hood C., Sackey S.S., Owusu A., Tatchie E., Nyarko S., Atieomo (2014). An assessment of heavy metal pollution in sediments of a tropical lagoon: a case study of the Benya Lagoon, Komenda Edina Eguafo Abrem Municipality (KEEA)—Ghana. J. Heal. Pollut..

[48] Essumang D.K. (2010). Distribution, levels, and risk assessment of Polycyclic Aromatic Hydrocarbons (PAHs) in some water bodies along the coastal belt of Ghana. Sci. World J..

[49] Dodoo D.K., Essumang D.K., Jonathan J.W.A. (2013). Accumulation profile and seasonal variations of polychlorinated biphenyls (PCBs) in bivalves Crassostrea tulipa (oysters) and Anadara senilis (mussels) at three different aquatic habitats in two seasons in Ghana. Ecotoxicol. Environ. Saf..

[50] Mahu E., Nyarko E., Hulme S., Swarzenski P., Asiedu D.K., Coale K.H. (2016). Geochronology and historical deposition of trace metals in three tropical estuaries in the Gulf of Guinea. Estuar. Coast Shelf Sci..

[51] Assiam B.Y. (2020).

[52] Dankwa H.R., Quarcoopome T., Owiredu S.A., Amedorme E. (2016). State of fish and fisheries of fosu lagoon, Ghana. Int. J. Fish. Aquat. Stud..

[53] Boateng I. (2012). An application of GIS and coastal geomorphology for large scale assessment of coastal erosion and management: a case study of Ghana. J. Coast Conserv..

[54] Osman A., Ko B., Mariwah S. (2016). International Journal of Disaster Risk Reduction Vulnerability and risk levels of communities within Ankobra estuary of Ghana.

[55] Aduah M.S., Warburton M.L., Jewitt G. (2015). Analysis of land cover changes in the bonsa catchment, Ankobra Basin, Ghana. Appl. Ecol. Environ. Res..

[56] Fianko Y., R J., Osae S., Adomako D., Adotey D.K., Serfor-Armah, Fianko J.R., Osae S., Adomako D., Adotey D.K., Serfor-Armah Y. (2007). Assessment of heavy metal pollution of the Iture Estuary in the central region of Ghana. Environ. Monit. Assess..

[57] Barry B., Obuobie E., Andreini M., Andah W., Pluquet M. (2005).

[58] Chapman J. (2007).

[59] Yankson K., Kendall M. (2001). https://fishcomghana.com/aquatic-biophysical-characteristics/a-students-guide-to-the-fauna-of-seashores-in-west-africa/.

[60] Lawson E.O. (2011). Physico-chemical parameters and heavy metal contents of water from the mangrove swamps of lagos lagoon, lagos, Nigeria. Adv. Biol. Res..

[61] Smith R. (2004).

[62] APHA/AWWA/WEF (2018). Standard methods for the examination of water and wastewater. Stand. Methods.

[63] Hach Company (2001).

[64] Dali G.L.A. (2023). Litter production in two mangrove forests along the coast of Ghana. Heliyon.

[65] Adjei-Boateng S., D, Obirikorang K.A., Amisah (2010). Bioaccumulation of heavy metals in the tissue of the clam Galatea paradoxa and sediments from the Volta Estuary, Ghana. Int. J. Environ. Res..

[66] Madkour D., A H., Obirikorang K.A., Amisah S., Otchere F.A., Adjei-Boateng (2011). Relationship between heavy metal concentrations in bottom sediments and the clam, Galatea paradoxa (Born 1778) from the Volta Estuary, Ghana. J. Environ. Protect..

[67] Soetan P.K., M O., Aggrey-Fynn J., Mensah (2021). 36th Annual Conference of Fisheries Society of Nigeria.

[68] Okyere I. (2018). Influence of diurnal tides and other physico-chemical factors on the assemblage and diversity of fish species in River Pra Estuary, Ghana. Trop. Ecol..

[69] Abdus-Salam N., Adekola F.A., Apata A.O. (2010). A physicochemical assessment of water quality of oil producing areas of ilaje, Nigeria. Adv. Nat. Appl. Sci..

[70] Rogers N.J., Urbina M.A., Reardon E.E., McKenzie D.J., Wilson R.W. (2016). A new analysis of hypoxia tolerance in fishes using a database of critical oxygen level (Pcrit). Conserv. Physiol..

[71] WRC (2003).

[72] Radojevic M., Bashkin V. (2006).

[73] Darko H.F., Ansa-Asare O., Paintsil A. (2013). A number description of Ghanaian water quality—a case study of the southwestern and coastal rivers systems of Ghana. J. Environ. Protect..

[74] Aheto D.W. (2011). Spatio-temporal analysis of two coastal wetland systems in Ghana: addressing ecosystem vulnerability and implications for fisheries development in the context of climate and land use changes. Arch. Appl. Sci. Res..

[75] Okyere I., Blay J., Aggrey-Fynn J., Aheto D.W. (2011). Composition, diversity and food habits of the fish community of a tropical coastal wetland in Ghana. J. Environ. Ecol..

[76] Faseyi C.A., Miyittah M.K., Sowunmi A.A., Yafetto L. (2022). Water quality and health risk assessments of illegal gold mining-impacted estuaries in Ghana. Mar. Pollut. Bull..

[77] National Estuarine Research Research (NERR) (1997). *North Carolina National Estuarine Research Reserve in Cooperation With NOAA-Washington,DC*, Estuary-Net.

[78] Nirmal Kumar J.K., George B., N. K R., Pr S., S V. (2009). Assessment of spatial and temporal fluctuations in water quality of a tropical permanent estuarine system - Tapi, west coast of India. Appl. Ecol. Environ. Res..

[79] Tufuor J.K., Dodoo D.K., Armah A.K., Darpaah G.A., Essumang D.K. (2007). Some chemical characteristics of the river PRA estuary in the western region of Ghana. Bull. Chem. Soc. Ethiop..

[80] Environmental Protection Agency (EPA) (1999).

[81] Bilotta G.S., Brazier R.E. (2008). Understanding the influence of suspended solids on water quality and aquatic biota. Water Res..

[82] Okyere I. (2019). Implications of the deteriorating environmental conditions of River Pra estuary (Ghana) for marine fish stocks. J. Fish. Coast. Manag..

[83] Iida C.L., Shock C.C. (2007).

[84] Sanders T., Laanbroek H.J. (2018). The distribution of sediment and water column nitrification potential in the hyper-turbid Ems estuary. Aquat. Sci..

[85] NOAA/EPA, Susceptibility and concentration status of Northeast estuaries to nutrient discharges: northeast Case Study, Part 3: Strategic Assessment of near Coastal Waters. Protection Agency,” United States (1988).NOAA/EPA (1988). Susceptibility and concentration status of Northeast estuaries to nutrient discharges: Northeast Case Study. Part 3: Strategic Assessment of near Coastal Waters. United States. National Oceanic and Atmospheric Administration & United States. Environmental Protection Agency. 50 pp.

[86] Coastal Resource Center-Ghana/Friends of the Nation (2010).

[87] Howell J.A. (2010). The distribution of phosphorus in sediment and water downstream from a sewage treatment works. Biosci. Horizons.

[88] (2011). Coastal Resources Center and Friends of the Nation.

[89] EPA (2023). *Water Quality Topics: Pathogens*.

[90] Balogun K., Ladigbolu I., Ariyo A. (2011). Ecological assessment of a coastal shallow lagoon in Lagos, Nigeria: a bio-indicator approach. J. Appl. Sci. Environ. Manag..

[91] Adam C., Yao Aristide K., Ebrima N., Tidiani K., Konan Yao Aristide C. (2019). Diversity and spatial variation of benthic macroinvertebrates in the River Gambia estuary, West Africa. Int. J. Fish. Aquat. Stud..

[92] Venekey V., Fonseca-Genevois V.G., Santos P.J.P. (2010). Biodiversity of free-living marine nematodes on the coast of Brazil: a review. Zootaxa.

[93] Dean H. (2008). Revista de Biología Tropical the use of polychaetes (Annelida) as indicator species of marine pollution: a review Revista de Biología Tropical. Rev. Biol. Trop..

[94] Mendes Y.A. (2021). Sedentary fish as indicators of changes in the river flow rate after impoundment. Ecol. Indicat..

[95] Woke G., Wokoma I. (2007). Effect of organic waste pollution on the macrobenthic organisms of Elechi creek Port Harcourt. African J. Appl. Zool. Environ. Biol..

[96] Woke G.N., Wokoma A.I.P. (2000). Effect oforganic waste pollution on the macrozoobenthic organisms of the elechi creek, port harcout. African J. ofApplied Zool. Environ. Biol..

[97] Lencioni V., Marziali L., Rossaro B. (2012). Chironomids as bioindicators of environmental quality in mountain springs. Freshw. Sci..

[98] Sharma K.K., Chowdhary S. (2011). Macroinvertebrate assemblages as biological indicators of pollution in a central himalayan river , tawi (J & K). Int. J. Biodivers. Conserv..

[99] Belan T.A. (2003). Benthos abundance pattern and species composition in conditions of pollution in amursky bay (the peter the great bay, the sea of Japan). Mar. Pollut. Bull..

[100] Bloor M.C., Banks C.J. (2006). An evaluation of mixed species in-situ and ex-situ feeding assays: the altered response of Asellus aquaticus and Gammarus pulex. Environ. Int..

[101] Nkwoji J.A., Ugbana S.I., Ina-Salwany M.Y. (2020). Impacts of land-based pollutants on water chemistry and benthic macroinvertebrates community in a coastal lagoon, Lagos, Nigeria. Sci. African.

[102] Onyena A.P., Nkwoji J.A., Chukwu L.O. (2021). Evaluation of hydrochemistry and benthic macroinvertebrates in chanomi creek, Niger delta Nigeria. Reg. Stud. Mar. Sci..

[103] Li Y. (2021). Variation in bacterial communities among stress-sensitive and stress-tolerant black tiger shrimp (Penaeus monodon) individuals. Aquacult. Res..

[104] Arslan N., İlhan S., Şahin Y., Filik C., Yilmaz V., Önturk T. (2007). Diversity of invertebrate fauna in littoral of shallow musaözü dam lake in comparison with environmental parameters. J. Appl. Biol. Sci..

[105] Bo T. (2007). Effects of clogging on stream macroinvertebrates: an experimental approach. Limnologica.

[106] Biology E., State C.R., State B. (2012). The composition , distribution and abundance of macroinvertebrates in the shores of the great kwa river , cross river state , south-east , Nigeria. Eur. Jounal Zool. Res..

[107] Turkmen G., Kazanci N. (2010). Applications of various biodiversity indices to benthic macroinvertebrate assemblages in streams of a national park in Turkey. Rev. Hydrobiol..

[108] Pielou E. (1966). The measurement of diversity in different types of biological collections. J. Theor. Biol..

[109] Kocataş A. (1992).

[110] Marques J.C. (2001). Diversity, biodiversity, conservation, and sustainability. Sci. World J..

[111] Mistri M., Fano E.A., Rossi G., Caselli K., Rossi R., Rossi R. (2000). Variability in macrobenthos communities in the Valli di Comacchio, northern Italy, an hypereutrophized lagoonal ecosystem. Estuar. Coast Shelf Sci..

[112] Kim H.C., Montagna P.A. (2012). Effects of climate-driven freshwater inflow variability on macrobenthic secondary production in Texas lagoonal estuaries: a modeling study. Ecol. Model..

[113] Tachet H., Richoux P., Bournaud M., Polatera P. (2010).

[114] Ertaş A., Yorulmaz B. (2021). Assessing water quality in the kelebek stream branch (gediz River Basin, west anatolia of Turkey) using physicochemical and macroinvertebrate-based indices. Aquat. Res..

[115] Andem A.B., Okorafor K.A., Eyo V.O., Ekpo P.B. (2013). Ecological impact assessment and limnological characterization in the intertidal region of calabar river using benthic macroinvertebrates as bioindicator organisms.

[116] Jun Y.C. (2011). Effects of land use on benthic macroinvertebrate communities: comparison of two mountain streams in Korea. Ann. Limnol..

